# Strong Ligand-Protein Interactions Derived from Diffuse Ligand Interactions with Loose Binding Sites

**DOI:** 10.1155/2015/746980

**Published:** 2015-05-04

**Authors:** Lorraine Marsh

**Affiliations:** Department of Biology, Long Island University, 1 University Plaza, Brooklyn, NY 11201, USA

## Abstract

Many systems in biology rely on binding of ligands to target proteins in a single high-affinity conformation with a favorable Δ*G*. Alternatively, interactions of ligands with protein regions that allow diffuse binding, distributed over multiple sites and conformations, can exhibit favorable Δ*G* because of their higher entropy. Diffuse binding may be biologically important for multidrug transporters and carrier proteins. A fine-grained computational method for numerical integration of total binding Δ*G* arising from diffuse regional interaction of a ligand in multiple conformations using a Markov Chain Monte Carlo (MCMC) approach is presented. This method yields a metric that quantifies the influence on overall ligand affinity of ligand binding to multiple, distinct sites within a protein binding region. This metric is essentially a measure of dispersion in equilibrium ligand binding and depends on both the number of potential sites of interaction and the distribution of their individual predicted affinities. Analysis of test cases indicates that, for some ligand/protein pairs involving transporters and carrier proteins, diffuse binding contributes greatly to total affinity, whereas in other cases the influence is modest. This approach may be useful for studying situations where “nonspecific” interactions contribute to biological function.

## 1. Introduction

Ligand interactions with proteins may be specific or nonspecific. Many ligands bind to proteins via tight, cooperative interactions, that is, “lock and key” mechanisms. However, other, looser interactions also occur and may have physiological significance (e.g., in multidrug resistance). These interactions can be modeled by molecular dynamics (MD) [1], but the timescales involved in modeling multiple on and off diffusion, especially in and out of solvent, may strain the current limits of the technology. Pure Monte Carlo methods can estimate binding to loose protein cavities but can be inefficient, given the rugged energy profiles of binding and the large binding space occupied by clashing between ligand and receptor.

The MCMC approach has been widely used in physics and statistics to determine the probability distribution of multidimensional processes. For example, the distribution of molecular interactions with DNA has been modeled using MCMC [2]. Modeling uncertainty and low probability states in protein structure prediction also benefits from MCMC and related approaches [3]. A common theme in many structural analyses is the tradeoff between entropic and enthalpic contributions to free energy [4].

In the MCMC process, the probability of a state being occupied (number of steps occupying the state/number of total steps) is proportional to the stationary probability distribution of the process. One major advantage of MCMC is that high probability regions of the distribution are sampled more than low probability regions (importance sampling), increasing efficiency for study of distributions that have extensive regions of low probability. Another advantage is that extensive theoretical and practical applications of MCMC methods show that they are extremely robust and flexible approaches to model probability distributions [5].

For a MCMC method to be applicable, a distribution must exhibit certain traits. In particular, the distribution must satisfy regularity criteria. The process must be ergodic, that is, be capable of returning to any given state. For ligand-receptor interaction, this requires that solvent regions be finite. The process must be irreducible; that is, all states must be reachable by a random walk. In practice, this means that either ligands must access a solvent region that permits all conformations or the process must be allowed to jump to all allowed states. For instance, a ligand unable to rotate in a given site must be given a statistically valid path to rotate, either in solvent or through jump diffusion. In some cases, diffusion of small molecules will not require extra techniques to achieve irreducibility in large-volume sites.

Ligand binding pockets in proteins are diverse in shape, depth, and size [6]. Though many binding sites exhibit specificity required for their biological function, other molecules require less specificity to fulfill their purpose. The bacterial drug efflux pumps, including RND transporters such as AcrB, serve to export multiple toxic substrates from the cell [7]. Some of these substrates make fairly well-defined contacts with the binding cavity of the pump [8] while others may not. Computational studies have contributed to our understanding of the basis of drug pumping [1, 9]. AcrB preferentially pumps hydrophobic molecules and the pump chamber is lined with phenylalanine residues. Another example of molecules binding diverse substrates is the several families of sterol binding proteins, which also bind other lipids. The human serum albumin protein binds an extremely diverse set of ligands through two pockets and some crevice regions. Pocket 2, which is shallow and solvent accessible, binds the sedative diazepam and the anesthetic halothane amongst many substrates. Drugs may compete with each other for binding to albumin, which suggests some specificity of binding, but could be due to nonspecific occlusion of a hydrophobic patch available for contact [10].

Here, we treat a ligand molecule as a MCMC process diffusing within pose space in a receptor site, with its probability density distribution determined by the *K*_*d*_ of interaction with the receptor given its positional and rotational state for relatively rigid molecules. We show, using the MCMC method, that, for large sites, such as those of the AcrB bacterial drug transporter, multiple states or binding poses contribute to binding efficiency. This approach may have application to modeling other macromolecular interactions such as DNA-protein and protein-domain interactions. This method is also relevant to a number of pharmacological analyses.

## 2. Methods

### 2.1. Model Systems

Systems for ligand/protein binding analysis were selected based on the potential for loose, nonspecific interactions. Each of these proteins had large binding regions which offered ample room for the ligand to bind in multiple conformations and at multiple sites within the binding region of the protein. The AcrB multidrug transporter of* E. coli* undergoes cyclic changes that first open a large ligand-binding cavity and then expel the contents outside the cell. The cavity is large enough that all known ligands can, in principle, adopt many conformations within the cavity. The pump is very nonselective, suggesting that efflux does not require interaction with a specific evolutionarily selected binding site. Human serum albumin is an abundant protein in blood that plays many roles, including the transport of fatty acids. It also plays important roles in pharmacology by absorbing diverse drugs and reducing their free concentration in the bloodstream. Human serum albumin has at least two large pocket domains on its surface. Steroid transporters, also in the bloodstream, can bind many hydrophobic compounds including diverse steroids. The steroid transporters have pockets larger than what would be required to bind a simple steroid. These model systems were studied using the known sites of drug interaction.

The open binding chamber of AcrB (PDB ID: 3AOD, chain A) was studied with four ligands. Toluene is a solvent pumped by the exporter [11]; skatole is a toxic hydrophobic molecule ubiquitous in the natural environment of* E. coli*; acridine orange is the dye first used to characterize the exporter; and minocycline is an exported antibiotic crystalized with AcrB in the PDB ID: 3AOD structure. Two sterol binding proteins were studied as well (PDB IDs: 1ZHY and 2A1B). The human serum albumin (HSA) protein has two canonical ligand binding pockets. Pocket 2, reported to bind diazepam and halothane, was studied (PDB ID: 1E7B, chain A) [10, 12].

### 2.2. The MCMC Process

To study ligand binding in multiple conformations, it was necessary to estimate the proportion of ligand binding in each position. A MCMC process was designed such that the stationary probability in pose space would equal the predicted distribution of ligands in a binding site. To ensure ergodicity the process was constrained to a sphere centered on the initial ligand binding pose. Trial MCMC processes were studied to determine the effect of windowing and various parameters on performance. The spatial scoring window was set, conservatively, at 2 angstroms, since that is a commonly accepted RMSD for significantly similar poses for ligands and synchronized with the poxel definition used (see below). Depending on the number of steps and alpha, the step distance was analyzed. In general, the time to equilibration for an MCMC process is best determined by experimentation. A step size, *α*, for translation of 0.9 angstroms and an *α* for rotation of 0.6 radians were used. An important consideration for importance sampling of a rugged distribution is the tradeoff between the density of sampling and number of steps. For most systems studied, less than 200,000 steps were required to populate the majority of poxels. Stochastic jump diffusion to a probable poxel every 1000 steps was used to ensure sampling of all poses.

### 2.3. Scoring Affinity

A soft Lennard-Jones scoring parameter was employed as is common in successful empirical scoring functions [13–15]. The Δ*G* of atomic interaction was set at 1 kcal/mol at the VDW distance for the atoms involved [16, 17]. The *K*_*d*_ for interaction was calculated as *K*_*d*_ = *e* ^Δ*G*/*RT*^ with the MCMC Metropolis transition determined by 1/*K*_*d*_. Δ*G* for molecules in solvent was set at 0. For some experiments AutoDock Vina scoring [18] was used as an alternative method with similar results. This MCMC method is compatible with any scoring function for ligand/protein interaction.

### 2.4. Definition of Pose Space

Rigid ligands can be positioned in 6-dimensional translational/rotational conformational pose space. Pose space was divided into “poxels” by analogy with 3-dimensional voxels. Poxels were placed 3 angstroms apart in *x*, *y*, and *z* dimensions and 51.4° apart in rotational dimensions. No torsional dimensions were included for the rigid molecules studied here. These poxels were large enough that a single poxel could accommodate most of the motions of known tight-binding ligand/receptor complexes such as those found in PDB ID: 2rnh and 4gid. Note that molecules in adjacent poxels occlude the space of each other but are conformationally distinct and make different contacts with the receptor.

### 2.5. Analysis of Diffuse Binding

The MCMC method for DBF calculation was programmed in a Perl script. The MCMC process was typically run for 100,000 steps which typically populated the average poxel with more than 20 process visits. The most visited pose (modal) was recorded and entropy enhancement (EE) was calculated as total MCMC steps/modal MCMC steps. In essence, EE is the proportion of the steps a ligand spent at sites other than the modal pose. The less time spent at the modal pose, the more time spent in other poses. EE can be used to calculate the overall *K*_*d*_ (adjusted for multiple poses) as

(1) $$\begin{array}{c} \text{Overall}\;\;K_{d}=\frac{\text{Modal}\;\;K_{d}}{\text{EE}}. \end{array}$$

EE can be useful for converting the *K*_*d*_ predicted for binding to the single best binding site into the overall binding, allowing for pose flexibility. For typical high-affinity ligand-receptor pairs, the number of poses in the site was 1 or 2 and the MCMC steps for the modal pose equaled or nearly equaled the total number of steps, producing an EE value of ~1 and an overall *K*_*d*_⋍ modal  *K*_*d*_.

The effective number of poses for each site (DBF) was also calculated. DBF factors that many poses have a probability >0, nonetheless, are enthalpically unfavorable and hence poorly populated. For equally populated poses, the total poses equal DBF. For a more typical Poisson distribution, where the mean number of visits equals the standard deviation of the number of visits, DBF is 0.5x number of poses. DBF is defined as

(2) $$\begin{array}{c} \text{DBF}=\frac{\text{NP}}{\left(\left({\text{Var}}_{\text{MV}}\right)/{\text{MV}}^{2}\right)+1}, \end{array}$$

where NP is the number of *P* > 0 poses, MV is mean visits to a poxel, and Var_MV_ is the variance of MV. DBF is equal to the number of permissible poses if all sites bind equally. However, DBF is equal to ~1.0 if only a single pose has substantial probability, even if many poses with a probability >0 exist. If binding affinity is distributed amongst sites with a Poisson distribution, DBF⋍0.5∗NP. DBF is dependent on both the number of possible poses and the distribution of the probability of those poses. A high DBF indicates that diffuse binding plays an important role in overall affinity of a protein for a ligand.

## 3. Results and Discussion

### 3.1. Binding to a Loose Site

**Figure 1:**
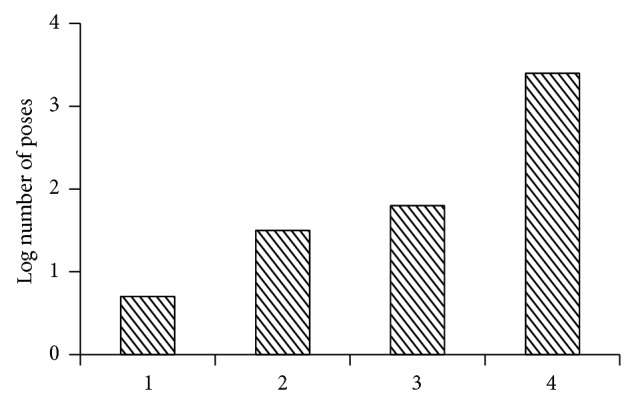
Number of poses in binding site for various ligands. (1) Beta-adrenergic receptor/carazolol (PDB ID: 2rhn_A), a typical tight-binding receptor/ligand complex; (2) AcrB/minocycline (28 heavy atoms); (3) AcrB/acridine orange (20 heavy atoms); and (4) AcrB/toluene (7 heavy atoms).

The AcrB binding site is approximately 2600 angstroms^3^ which is about twice the volume of the typical antibiotics that it pumps and about 12 times the volume of toluene. Analysis of binding of acridine orange (originally used to discover the AcrB gene) and other ligands showed that they could bind in several conformations (Figure 1 and Table 1).

DBF values for various ligands binding to the cavity of the AcrB multidrug pump varied greatly. Not surprisingly, the smaller ligands had higher DBF values than the larger ones (Table 1) indicating that diffuse binding contributes more to their overall affinity. Minocycline, which has been crystalized with AcrB in a single pose, had effectively 1-2 poses in this analysis as well, though a number of unfavorable poses had a probability >0. The absolute DBF value may be slightly inflated since the poxel dimensions were conservatively defined and even a relatively tight-binding molecule often has some freedom to move within its site. DBF comparisons between ligands may be more useful for some purposes. Toluene, a substrate that has been shown to be pumped by this transporter had a very high DBF, approximately 100 times that of minocycline. As discussed below, a mixture of many low-affinity sites and one higher affinity site seems to contribute to AcrB affinity for toluene. Acridine orange, the substrate originally used to characterize AcrB, had a low DBF, but more than one pose seemed to contribute to its binding. Skatole, a molecule toxic to* E. coli* but ubiquitous in its environment, was intermediate between acridine orange and toluene. Using empirical scoring, skatole had a DBF more similar to toluene, suggesting that for some ligands the choice of scoring function might be significant. The difference was due to the presence of a single high-affinity AcrB/skatole poxel in the atomistic analysis that was absent in the empirical analysis. However, both scoring methods predicted that most of skatole affinity involved diffuse binding. The other tested ligands had similar DBFs with both scoring methods. For the small ligands, substantial enthalpy-entropy compensation was possible, suggesting that the potential to bind with many poses contributed to efficiency of pumping these molecules. Similar effects may influence patterns of multidrug resistance in other systems [19]. It should be noted that the increased affinity predicted for small molecules such as toluene and skatole is not purely an entropic effect. The molecules have more freedom to move but are also able to make enthalpically favorable (though weak) contacts with the pump in these alternative poses. These loose configurations may resemble other examples of ligands binding in sites larger than what the molecules require. For example, in virtual screening, decoy molecules may appear to be able to bind sites that do not fit well [19].

### 3.2. Visualization of Relative Poxel Probability

**Figure 2:**
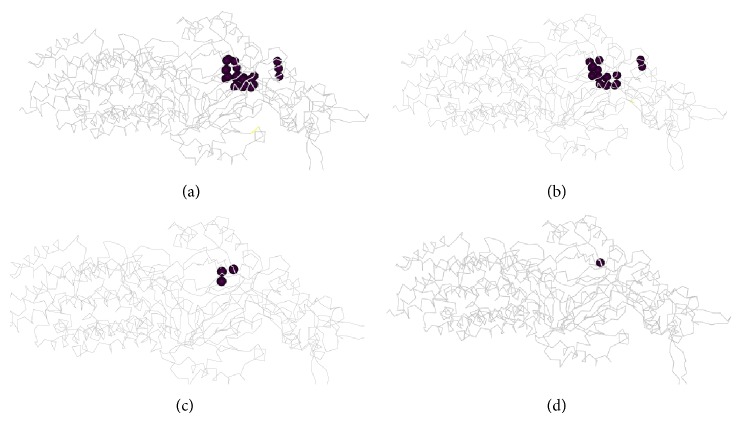
Toluene binding sites in AcrB. The centroids of the sites are shown. Rotationally equivalent poses may occupy a single site. (a) All pose sites are shown. (b) Sites contributing 52% of total binding are shown. (c) Poses contributing 15% of binding are shown. (d) A single pose contributing 10% of binding is shown. Though a relatively high-affinity site exists, low-affinity sites make up the bulk of binding probability.

Populated poxels (*P* > 0) were visualized for toluene (Figure 2). Poxels with some probability of being populated were not clustered, indicating that several sites in the large cavity contribute to affinity. The distribution of MCMC visits to poxels was determined. The distribution of poxels contributing about 50% of total affinity was also dispersed (Figure 2). However, one pose alone contributed about 10% of the total probability and could be considered a relatively high-affinity site. If crystallization of AcrB with toluene was successful one might predict that this site would appear as the site of toluene interaction. However, the analysis here suggests that most of the experimentally determined affinity would be due to diffuse, low-affinity interactions. The poxels of minocycline/acridine orange clustered at the region of the minocycline site in the crystal structure PDB ID 3AOD. Skatole poxels were scattered, in a Poisson-like distribution similar to that of toluene.

**Figure 3:**
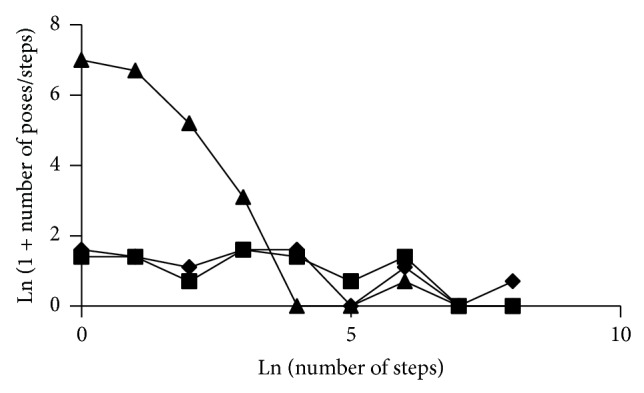
Specific versus nonspecific binding. The number of steps per pose is plotted versus the number of poses with a specific number of steps. A high value on the vertical axis indicates many low-affinity poses. A high value on the horizontal axis indicates a few high-affinity poses. Triangles, AcrB/toluene; squares, AcrB/acridine orange; diamonds, AcrB/minocycline. The graph for skatole was nearly identical to the line for toluene except for a single higher affinity site and is omitted for clarity. See text for details.

Overall, the probability distribution of toluene in AcrB resembles a Poisson distribution of site occupancy (Figure 3) with the mean number of poxels occupied increasing as affinity decreases. Skatole, which chemically resembles toluene, had a nearly identical distribution to toluene, though its DBF was lower because of a single pose with higher affinity than the highest affinity site of toluene. Significantly, despite the existence of higher affinity sites, most of the binding of toluene and skatole is still derived from interaction with multiple low-affinity sites. In contrast, the affinity of both acridine orange and minocycline is dominated by one or a few poses, with only smaller, less significant, contributions from alternative sites (Figure 3 and Table 1). The DBF values for toluene were roughly 100-fold higher than those for minocycline, indicating a much greater role for diffuse binding in toluene binding than for minocycline binding. Thus small ligands binding to the AcrB site derive an affinity benefit from the ability to bind weakly in many conformations, while larger ligands benefit by binding in only a few conformations, but making more receptor/ligand contacts in each conformation. Acridine orange and minocycline also have fewer poxels that do not clash with the AcrB pump chamber than toluene or skatole.

### 3.3. Other Nonspecific Complexes

As a comparison with AcrB, two sterol binding proteins were analyzed. These molecules, oxysterol carrier protein and cinnamomin, both bind to a number of sterols with little specificity. They have no sequence or structural similarity. Both bound sterol with only a single pose showing high probability, though other poses did have a probability >0 (Table 1). This result suggests that the flexibility and looseness of their respective binding pockets serve mostly in accommodating ligand diversity and not increasing ligand affinity for the molecules studied.

Human serum albumin (HSA) has been extensively studied for its ability to bind naturally occurring hydrophobic molecules such as fatty acids and also drugs. Competition studies and X-ray crystallographic studies suggest that two large pockets are involved in at least some of the binding [10]. When binding of halothane and diazepam, which bind pocket 2, was studied by the MCMC method considerable pose diversity appeared to be present within the known binding pocket (Table 1). These molecules, both by visual inspection and docking studies with AutoDock Vina, did not have binding sites with the characteristics of known high-specificity sites. They appeared to bind in many poses to the hydrophobic pocket region 2 of HSA and occlude the site, competing in that way with other substrates. As with toluene binding to AcrB, a few higher affinity sites were present, but the overall predicted affinity was dominated by a large number of low-affinity interactions. In contrast, binding of carazolol to the beta-adrenergic receptor (a classic “lock and key” tight interaction) yielded a DBF of only 1.8.

## 4. Conclusions

The MCMC method provides an approach to conceptualize and study diffuse binding interactions that have heretofore been relegated to the area of “nonspecific” interactions. These types of interactions are likely to play important roles in some ligand binding, DNA-protein interactions and in domain interactions in proteins, especially the interactions of intrinsically disordered domains. In some cases this analysis has shown that nonspecific interactions might also contribute to overall affinity of ligands. In other cases, apparently loose fitting ligands actually bind with a single effective pose. MCMC methods complement molecular dynamic approaches and allow modeling of interactions that occur over relatively long time periods. The MCMC approach is reasonably fast and statistically rigorous and thus provides a link between physics-based methods that attempt to model actual molecular behavior and algorithmic methods that seek to efficiently provide a single best binding conformation.

## Figures and Tables

**Table 1:** Relative contributions of multiple poses to predicted affinity.

Complex	Poses	DBF	EE
*Spatial fit scoring *			
AcrB/minocycline	36	5 ± 2	2.5 ± 0.8
AcrB/acridine orange	68	14 ± 4	6.9 ± 1.2
AcrB/skatole	1494	57 ± 31	9.7 ± 5.7
AcrB/toluene	2429	661 ± 139	53.7 ± 10.1
OSBP/cholesterol	10	1.3 ± 0.3	1.1 ± 0.2
Cinnamomin/ergosterol	7	2.2 ± 1.1	1.7 ± 0.4
HSA/diazepam	5050	2298 ± 1703	435 ± 333
HSA/halothane	6897	4319 ± 1626	1630 ± 695
BAR/carazolol	3	1.8 ± 0.9	1.5 ± 0.4
*Empirical scoring *			
AcrB/minocycline	52	8.5 ± 2.0	4.1 ± 1.7
AcrB/acridine orange	81	5.7 ± 3.1	3.3 ± 2.3
AcrB/toluene	1965	726 ± 67	107 ± 10
AcrB/skatole	1637	526 ± 58	76 ± 9

Abreviations: OSBP, oxysterol binding protein; HSA, human serum albumin; BAR, beta-adrenergic receptor.

“Poses”, refers to number of possible (*P* > 0) poses in binding region analyzed.

Analyses performed in triplicate with runs of 100,000 MCMC steps.
